# Fifth Biennial Report of the Board of Trustees of the Illinois State Hospital for the Insane

**Published:** 1857-02

**Authors:** 


*Fifth Biennial Report of the Board of Trustees of the Illinois State Hospital for the Insane.* December, 1856. Charles Scott, Book and Job Printer, Chicago.

This is a neatly-printed pamphlet of fifty-two pages, containing the reports of the Board of Trustees, the Treasurer, and the Superintendent of our State Hospital for the Insane, at Jacksonville. From the Report of the Superintendent, Dr. Andrew McFarland, we select some items of interest to the profession.

The following will show the number treated in the Institution during the last two years, with the results:—

'There were in the Hospital, at the date of the last report (December 1, 1854,) one hundred and sixty-six patients. Three hundred and two have since been received; making the whole number in the Institution during the two years, *four hundred and sixty-eight.* Two hundred and fifty-four have, in the mean time, either been discharged or have died; leaving now in the Hospital two hundred and fourteen. *Ninety-four* of these are males, and *one hundred and twenty* are females. Of those discharged, one hundred and eighteen were, in our opinion, *recovered.* Fifty-six were in a decidedly *improved* condition; but discharged short of full recovery, for reasons considered sufficient. Thirty-six were discharged by the Trustees, in obedience to the provisions of Section Fourteen, of the Statute of 1853—they being considered incurable, but perfectly harmless. Twenty-one were discharged at the request of their friends, in a stationary or wholly unimproved state. Twenty-three have died from causes hereafter to be stated.'

For the guidance of those who are interested in procuring admission for patients, the following paragraph may be important:—

'In passing this portion of the Report, it is proper to call the attention of county authorities to an evil which we fear acts prejudicially upon those having a claim upon the Hospital, no less than on the Institution itself. In some instances, those having insane friends have applied for information in regard to their admission to the Hospital to the authorities of the county in which they reside, and have been met by the reply, that the Institution could only admit a given number from each county, that the quota from their own was already made up, and that no more could be admitted. We have cause to suspect, that, in some instances, where this reply has been given, those in the Institution from the county have been the paupers from its alms-house or jail, while those seeking in vain for admission were the ones whose means were taxed to support the Institution. There is no remedy for this but in the fidelity of those who are supposed to be best informed in relation to the Hospital. The truth is, that, full as the Institution always is, no case affording any just hope of cure or material relief has ever been denied admission.'

From the Superintendent's brief but interesting comments on the supposed causes of insanity, we make the following extracts:

'Another result of continued observation among the insane, is, that special or exciting causes have less weight in the production of mental disease in the mass of cases, than such as are predisposing or constitutional. What is frequently given by the unskilled observer as the cause of the disease, is merely one of its accidental manifestations.

'An individual, for instance, among whose near or remote ancestry insanity has been recognized, changes, without apparent cause, the ordinary course of his life, or becomes marked by peculiarities not before known to exist. These peculiarities may have a religious cast. Why that aspect is more commonly presented than others, will be hereafter considered. From being careless in spiritual matters, he applies himself to the perusal of the Bible, to conversation on religious subjects, and attendance on religious worship. This, at first, attracts no especial attention. Gradually a fervor is infused into his exercises, not wholly natural or temperate; his vigils grow protracted; his food and sleep are neglected; and the most fearful apprehensions of the torments of a coming judgment fill his soul with terror. His exhortations grow vehement, the consistency and connection of his ideas finally break down, and his reason totally disappears in the mental chaos which follows. This is acute mania in its completest form.

'The case is committed to us as one unquestionably caused by "religious excitement"—yet nothing can be less conclusive: the religious cast infused into all the outward manifestations of the insanity is simply accidental.

'There appear to be two reasons why the mental manifestations of the insane have so frequently a religious tinge. One is, that preceding every attack of insanity from constitutional causes, there seems to be a period when most individuals have an indistinct consciousness that something unusual is about to happen. The mind dimly and fearfully apprehends the storm that is approaching. Reason, trembling with these fearful premonitions, seizes for support on that latent religious sentiment which lies in every human breast; and when the storm really bursts in its fury, this sentiment remains prominent in all the ruin that follows. Another reason seems to be, that the insane mind has a natural affinity for the unseen and mysterious. The talk of the insane man is often of "spirits," "heavenly telegraphs," "mesmerism," "magnetism"—of subjects lying on that debatable ground where natural science loses itself in the mythical. Nothing is so natural, then, as that the great "Mystery of mysteries" should, more than all else, fill the clouded and contracted circle of the maniacal vision. That the truths of the Christian religion, brought before the attention by any ordinary induction, ever produced insanity in a mind of healthy constitution, is supported by no valid experience. If the conception of religious truth produced insanity, by itself, we should infer that those great, comprehensive, and impassioned minds which, in Edwards and Whitfield, glowed with such religious fire, would supply cases in proof. On the contrary, those brought to us as of that character, are quite as frequently like him

> "Who never had a dozen thoughts in all his life,
> And never changed their course."

'On the whole, while willing to allow that the well-balanced mind is frequently thrown from its equipoise by specific causes, in the great number of cases, long antecedent preparatory influences have been at work. That these influences are depressing in most if not all cases, is proved by the success which attends the modern treatment as distinguished from that in vogue three-fourths of a century since. The general and local depletions, the counter-irritants, the spare diet, and the routine once prescribed under the general phrase of "antiflogistic regimen," have long since yielded to a method diametrically opposite. Even cases which bear on their surface the tokens of sthenic action are now met with constant success by a liberal diet and properly graduated stimulants. We allude to this more particularly because we yet occasionally receive patients who have, to quote a descriptive letter recently received with one of them, "been well bled and blistered, *but without apparent effect.*"

'The peculiar pathological condition of the system, known among medical men as the "puerperal state," has been an extremely prolific cause of insanity during the period which this report covers. This may be partly an accident of the time, though we are satisfied that insanity from causes incident to the child-bearing state is more frequent in the circle from which the patients in this Institution are derived than those contiguous to most other hospitals whose reports reach us. Marriages entered into before the physical system has reached its full maturity; the great dearth of means of sufficient intelligence to render proper assistance at such a critical period; the discomforts attending a sparse population; and the other deprivations of frontier life, are sufficient to account for the prominence of this among the causes enumerated in our table. The number of chronic cases of this character has somewhat surprised us.

'The following case (not a supposed one) will serve as the type of several that have come under our notice:—

'Mrs. ———, three days after accouchment, imprudently left her bed and sat by the fire, in the only room in which she at that time lived, while the floor was being washed. The next day she appeared uncommonly cheerful and talkative, and left her bed, stating that she was well, and that no further attendance was necessary. From that time it was observed, by those intimate with her, that her former character and disposition appeared changed. She became capricious in her attachments, jealous of the motives of those about her, treated some of her children with ungrounded aversion, and manifested other indications of an impaired moral sensibility. There was a distinct periodicity in the exhibition of these symptoms. At length, after several years continuance of this latent mental disease, it developed itself into an attack of violent mania, during which she was brought to the Hospital, where inquiry revealed the facts above noted. Many such cases doubtless escape the attention of medical men, but are the occasion of untold distress in the family in which they may occur. The forms of insanity known in the books as *Oikeiomania* (morbid state of the domestic relations), and *Kleptomania* (unnatural propensity to steal), are, doubtless, less frequently traceable to this particular cause. Those conversant with the doings of the New York police courts will remember the very recent case of a lady, from a high position in society, who was arraigned for theft. Among the reasons urged in mitigation of sentence, it was stated, that this was her first offense, and that she had a babe at home but *nine weeks old.* The last fact furnishes abundant explanation. Cases of such a kind are often of life-long duration—riving asunder the family tie in many instances, unless, as in the case first quoted, it changes into the form of open and unequivocal insanity.'

We had marked several other paragraphs for quotation, but have not space for them in this number of the *Journal;* and we will only add that the report, as a whole, is highly interesting and instructive, and exhibits abundant evidence that the Hospital for the Insane is not only one of the noblest charities of the State, but is also under the immediate control of most able, efficient, and faithful officers.

